# Removal of Diclofenac, Paracetamol, and Carbamazepine from Model Aqueous Solutions by Magnetic Sol–Gel Encapsulated Horseradish Peroxidase and Lignin Peroxidase Composites

**DOI:** 10.3390/nano10020282

**Published:** 2020-02-07

**Authors:** Ievgen V. Pylypchuk, Geoffrey Daniel, Vadim G. Kessler, Gulaim A. Seisenbaeva

**Affiliations:** 1 Department of Molecular Sciences, Swedish University of Agricultural Sciences, 750 07 Uppsala, Sweden; vadim.kessler@slu.se; 2 Chuiko Institute of Surface Chemistry of National Academy of Sciences of Ukraine, 17, General Naumov Street, 03164 Kyiv, Ukraine; 3 Department of Biomaterials and Technology, Swedish University of Agricultural Sciences, Box 7008, 750 07 Uppsala, Sweden; geoffrey.daniel@slu.se

**Keywords:** sol–gel silica encapsulation, horseradish peroxidase, lignin peroxidase, enzymatic drug decomposition, magnetic nano-carrier, diclofenac, paracetamol, carbamazepine

## Abstract

Sustainable and green synthesis of nanocomposites for degradation of pharmaceuticals was developed via immobilization and stabilization of the biological strong oxidizing agents, peroxidase enzymes, on a solid support. Sol–gel encapsulated enzyme composites were characterized using electron microscopy (TEM, SEM), atomic force microscopy, FTIR spectroscopy, and thermogravimetric analysis. Horseradish peroxidase (HRP) and lignin peroxidase (LiP) were adsorbed onto magnetite nanoparticles and sol–gel encapsulated in a surface silica layer. Encapsulation enhanced the stability of the biocatalysts over time and thermal stability. The biocatalysts showed appreciable selectivity in oxidation of the organic drinking water pollutants diclofenac, carbamazepine, and paracetamol with improved activity being pharmaceutical specific for each enzyme. In particular, sol–gel encapsulated LiP- and HRP-based nanocomposites were active over 20 consecutive cycles for 20 days at 55 °C (24 h/cycle). The stability of the sol–gel encapsulated catalysts in acidic medium was also improved compared to native enzymes. Carbamazepine and diclofenac were degraded to 68% and 64% by sol–gel LiP composites respectively at pH 5 under elevated temperature. Total destruction of carbamazepine and diclofenac was achieved at pH 3 (55 °C) within 3 days, in the case of both immobilized HRP and LiP. Using NMR spectroscopy, characterization of the drug decomposition products, and decomposition pathways by the peroxidase enzymes suggested.

## 1. Introduction

Worldwide urbanization and population increase and associated expansion in the use of pharmaceuticals is driving the search for new materials to insure efficient and rapid purification of both drinking water and wastewaters. Apart from the widespread problem of heavy metal pollution aggravated by warmer weather and increased acidity [1], there is a growing worldwide problem of water pollution by pharmaceuticals. It is well-known that the content of persistent organic pollutants such as pharmaceuticals [2], personal care products [3], and their metabolites are increasing due to human activity. For example, in wastewater treatment plant (WWTP) effluents, the concentration of carbamazepine (CBZ) has been reported as reaching 0.95 μg/L, and 0.99 μg/L for diclofenac [4]. In particular, the anti-inflammatory drug diclofenac has been detected in surface, ground and drinking waters in the range of ngL^−1^ to µgL^−1^ in Switzerland [5], Spain [6], Sweden [7], and in the Baltic region [8]. Even other pharmaceuticals—such as ibuprofen, carbamazepine, tramadol, naproxen, and oxazepam—were recorded recently in drinking waters in Sweden [7].

What is surprising, efficiency of conventional WWTPs against micropollutants was close to 0%, but with high variability for individual compounds [9]. Better results in pharmaceuticals removal were achieved using nanofiltration followed by a carbon filter adsorption (90% median treatment efficiency). Despite apparent high efficacy, removal of pharmaceuticals by adsorption has some disadvantages, such as low adsorption capacity, and low selectivity. Also, the principal challenge in the removal of persistent organic pollutants and pharmaceuticals is that conventional sorbents are not efficient against the nano concentrations recorded and the need for extremely long time for removal. For example, biochars proposed for home use require up to 22 weeks for effective carbamazepine removal [9,10]. In addition, disposal of adsorbents contaminated by pharmaceuticals is in question. 

Besides adsorbents, other technologies for pharmaceutical removal, for example by ozone and UV, are already available for public. For instance, pilot-scale tests were run with ozonation for reduction of 24 pharmaceuticals at 10 full-scale wastewater treatment plants in southern Sweden with reasonable level reduction of pharmaceuticals reaching on average 65–88% [11]. UV-treatment chambers were installed in Stockholm and have a capacity to deliver clean water to about half a million people [12]. In model investigations, authors reached 27% of diclofenac removal by UV [13] at pH = 7.0. Also, close to 90% of CBZ removal was reached by O_3_, H_2_O_2_ and UV [14]. Nevertheless, common features during UV-treatment and ozonation are high energy consumption and production of potentially toxic and even carcinogenic byproducts [15].

In this respect, due to its high selectivity and performance, enzymatic water treatment was proposed as an attractive alternative solution [16,17,18]. 

For example, crude lignin peroxidase (LiP), from the white rot fungus *Phanerochaete chrysosporium* was studied for in vitro degradation of CBZ and diclofenac. LiP was found to completely degrade diclofenac at pH 3.0–4.5 with 3–24 ppm H_2_O_2_, while the degradation efficiency of CBZ was mostly below 10% [19]. In another study, the effective elimination of CBZ (60–80%) was stable in a fungal bioreactor for around 100 days. The main catalysts in that fungal bioreactor were lignin peroxidase, manganese peroxidase, and laccase [20,21]. 

**Figure 1:**
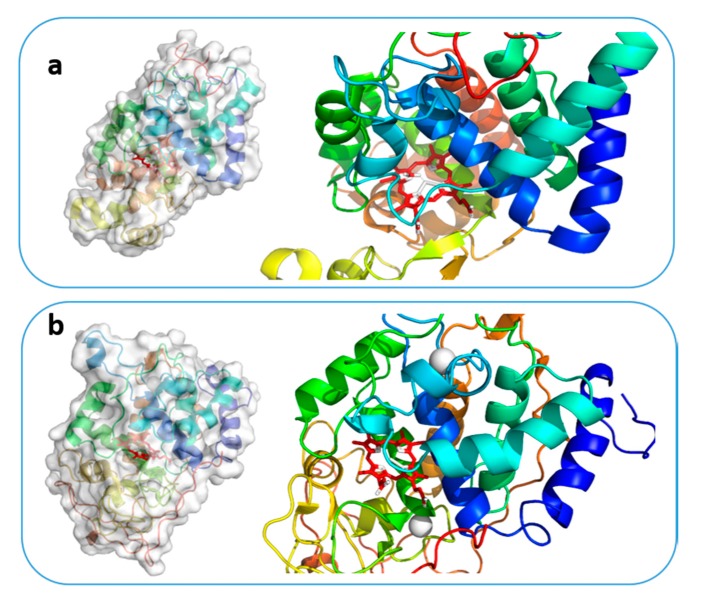
Molecular structure of HRP (**a**) and LiP (**b**) (left) with detailed view of their active sites. The heme-unit is highlighted with red color. The image has been produced in this work, applying PyMol software [31].

Horseradish peroxidase has also been evaluated for removal of drugs such as diclofenac [22] (native HRP, up to 47% removal) and paracetamol (immobilized HRP, 83.5% removal) [23]. Carbamazepine and diclofenac pollution and their removal by wastewater plants was reviewed in [24]. Enzymatic removal of urea, diclofenac, and acetaminophen by laccase and urease in the presence of heavy metals were addressed in our previous studies [25,26]. The molecular structures of HRP (a) and LiP (b) are presented in Figure 1 below. LiP possesses a higher theoretical redox potential than HRP [27].

Immobilization on a solid carrier generally increases thermal stability of enzymes and their number of operation cycles, reducing thus the costs for industrial use. Besides methods of immobilization, it is well-known that encapsulation of enzymes into silica matrices can also increase their thermal stability and resistance [28,29,30]. 

This manuscript was conceived due to lack of information about immobilized LiP enzyme performance. Mostly, only native LiP was reported for pharmaceuticals degradation [28,32,33,34,35], except work [28].

Based on the above considerations, we supposed that a combined approach consisting of immobilization of enzymes followed by encapsulation into a silica matrix should increase their thermal- and operational stability. In the present contribution, new enzymatic nanocomposites based on prior immobilized HRP and LiP and subsequent encapsulation into silica a matrix were developed and applied in a green process for common drug pollutant decomposition and removal. Our idea was to preserve beneficial conformation of the enzymes on a magnetite surface, covering it by silica shell, providing physical barrier to enzyme shielding from mechanical damage and interaction with highly molecular compounds. 

## 2. Materials and Methods

The following reagents were used in the applied synthetic procedures: tetraethoxysilane (TEOS, Si(OC_2_H_5_)_4_, Sigma-Aldrich Sweden AB, cat. no. 8.00658 98%, Stockholm, Sweden); iron (II) chloride tetrahydrate (FeCl_2_·4H_2_O, Sigma-Aldrich Sweden AB, cat. no. 380024 97%, Stockholm, Sweden); iron (III) chloride hexahydrate (FeCl_3_·6H_2_O, Sigma-Aldrich Sweden AB, cat. no. 31232-M 98, Stockholm, Sweden); sodium acetate tri-hydrate (VWR International AB, product: 27652.232, Stockholm, Sweden); glacial acetic acid (Scharlau, HPLC grade, Barcelona, Spain); hydrogen peroxide (H_2_O_2_, 36% in water, Sigma-Aldrich Sweden AB, Stockholm, Sweden); acetaminophen (Sigma-Aldrich Sweden AB, powder 98.0–102.0%, Stockholm, Sweden); diclofenac sodium salt (Sigma-Aldrich Sweden AB, product number: D6899, Stockholm, Sweden); ethanol (96%); 0.1 M HCl; 0.1 M NaOH; 0.06 M phosphate buffer (pH 7.0); horseradish peroxidase, (Sigma-Aldrich Sweden AB, lyophilized, powder, beige, ~150 U/mg, Stockholm, Sweden); lignin peroxidase (Sigma-Aldrich Sweden AB, powder, slightly beige, >0.1 U/mg, Stockholm, Sweden); ABTS (2,2′-azino-bis(3-ethylbenzothiazoline-6-sulphonic acid), Sigma-Aldrich Sweden AB, Stockholm, Sweden). 

### 2.1. Magnetite Synthesis

Magnetite was prepared by co-precipitation of iron (II) and iron (III) chlorides with ammonia solution under a nitrogen atmosphere [36]. For that, 24g of ferrous chloride (FeCl_2_) and 48 g of Ferric chloride (FeCl_3_) were dissolved in 1l of deionized water. This solution was added dropwise to 250 mL of ammonia solution (NH_4_OH, 25% in water). Black precipitate was collected and washed several times by distilled water to pH = 7. The obtained Fe_3_O_4_ particles were spherical with an average diameter of about 14 ± 3 nm, and a specific surface area of about 96 m^2^/g. 

### 2.2. Immobilization of Enzymes and Their Encapsulation into Silica Matrix

200 mg Fe_3_O_4_ was suspended in 50 mL of water/ethanol (1:1, v/v) mixture and then 5 mg of the enzyme dissolved in 5 mL of phosphate buffer was added to the magnetite suspension. The enzyme was left to absorb for 1.5 h, then 0.2 mL of 1% NH_4_F in water was added. Finally, 4 mL TEOS in 5 mL EtOH was added over 30 min. After 5 h, the solution became viscous and transformed into a gel. The mature gel was washed by EtOH and water. The composites were freeze-dried overnight. 

### 2.3. ABTS Oxidation Test and Enzyme Activity

One unit (U) of enzyme activity was defined as the amount of enzyme required to oxidize 1 µmol of ABTS per minute with free enzyme activities expressed in UL^−1^. Triplicate measurements were made for each assay of enzyme activity. ABTS was prepared as a 0.2 mM solution in acetate buffer at pH 5.0. With each cycle of oxidation, 3 µL of 3.6% H_2_O_2_ and 0.997 mL of ABTS was added to interact for 24 h with the composite powder at defined temperature. Composites were separated by magnet within 1 min. After separation, the ABTS solutions were filtered through 0.2 µm cellulose membranes in order to separate composite particles. ABTS concentration was measured at 420 nm in a quartz cuvette with a light path length of 1 cm.

### 2.4. Drug Solutions

All drugs solutions were prepared in water or acetate buffer with corresponding pH. Initial concentrations were 17.6 µg/mL for CBZ, 100 µg/mL for paracetamol and 50 µg/mL for diclofenac.

### 2.5. Drugs Decomposition by Native Enzymes

Initial enzyme activity was measured after by ABTS test (10 µL of enzyme solution + 3 µL of H_2_O_2_ (3.6%) + 1 mL ABTS in sodium acetate buffer) after reacting for 10 min. Then, 1 mL of crude enzyme solutions in phosphate buffer, (HRP and LiP) with C = 0.06 mg/mL (corresponding to the mass of enzyme in composite), were mixed with 1 mL of 0.01 mg/mL of paracetamol solution, or diclofenac solution or with 1 mL of 17 mkg/mL of carbamazepine solutions, following with 3 µL of H_2_O_2_ (3.6%) addition. Solutions were reacting for 5 days, then filtered through 0.2 µm cellulose membrane filters.

### 2.6. Drugs Degradation by Non-Encapsulated Enzymes

Experiments were done with and without enzymes; experiments were multiplicated and ran in a few cycles. 1 mL of drug solution in corresponding buffer was added to 30 mg of magnetite-enzyme sample (200 mg Fe_3_O_4_ was suspended in 50 mL of water/ethanol (1:1, v/v) mixture and then 5 mg of the enzyme dissolved in 5 mL of phosphate buffer was added to the magnetite suspension; the enzyme was left to absorb for 1.5 h) under vigorous shaking with following 3 µL of H_2_O_2_ (3.6%) addition.

### 2.7. Drug Decomposition by Hybrid Composites

1 mL of drug solution in corresponding buffer was added to 30 mg of sol–gel sample under vigorous shaking, followed by the addition of 3 µL of H_2_O_2_ (3.6%). Samples were thermostated at a certain temperature and time. 

### 2.8. Enzyme Leaching from Sol–Gel Encapsulated Composites

30 mg of sample has been shaking at 55 °C for 24 h in deionized water. After composite decantation by magnet, solutions were filtered through 200 µm membrane filter and measured by UV–Vis at λ = 280 nm. Then, ABTS was added and solution was kept for 24 h at 55 °C to react. Activity was measured at λ = 420 nm.

### 2.9. NMR Experiments

500 µL of a sample solution after removal of the nanocomposite was filtered through 0.2 µm cellulose membranes and mixed with 55 µL of D_2_O. 600 MHz Bruker Avance Cryoprobe instrument was used.

### 2.10. UV–Vis Spectroscopy

Concentrations of drugs were measured using UV–Vis spectroscopy at λ = 243 nm for the paracetamol, λ = 273 nm for the diclofenac, and at λ = 280 for CBZ. All solutions were filtered through 0.2 µm cellulose membrane filters in order to separate composite particles. Perkin Elmer Lambda 2 instrument was used.

### 2.11. SEM

Micrographs of samples were obtained on an Hitachi TM-1000 environmental scanning electron microscope. Samples were mounted on specimen stubs coated by carbon tabs and viewed at 15 kV. 

### 2.12. FTIR

FTIR spectra of all samples were recorded as KBr pellets on a Perkin-Elmer Spectrum 100 instrument. A total of eight scans were carried out on wavenumbers from 400 cm^−1^ to 4000 cm^−1^, in transmittance mode. All spectra were smoothed and their baselines corrected.

### 2.13. AFM

Surface structures of samples were studied using a Bruker FastScan Bio Atomic Force Microscope (AFM) in ScanAsyst mode. Wet samples were air-dried for 2 days prior to investigation. For native enzymes, samples were prepared by depositing 2 µL of 0.06 mg/mL solution on a mica plate. Composite samples were prepared by deposition of 2 µL of 0.001 mg/mL suspension on mica plate.

### 2.14. Transmission Electron Microscopy

TEM observations were made using a Philips CM12 instrument (Philips, Sweden) operated at 100 kV.

### 2.15. TGA

Thermogravimetric analysis was carried out in air with a heating rate of 10 °C min^−1^, using a Perkin-Elmer Pyris 1 instrument. 

### 2.16. Differential Scanning Calorimetry (DSC)

DSC was performed with Mettler-Toledo DSC 820instrument. The method consisted in heating of the sample in 40 µL Al crucible at a rate 8 °C/min and airflow rate 50 mL/min up to 600 °C.

### 2.17. Statistical Analysis

Statistical analysis was performed using Origin 2017 software. Error bars in the graphs were expressed as mean square deviation of the data. 

## 3. Results and Discussion

Some materials, due to their surface charge and adsorbed counterions, provide special ‘microenvironment’ for enzymes, thus increasing their activity. For instance, modeling for *Trametes Versicolor* laccase revealed higher enzyme performance when its immobilized on positive surfaces [37]. Surface of magnetite has proved to be such an “enhancer” for laccase enzyme [26].

In the present work, we applied a combined approach, consisting of adsorption of the enzyme on magnetite (Fe_3_O_4_) surface in a first step, followed then by formation of a SiO_2_ shell using a sol–gel method. Magnetite was used as an active surface for freezing enzyme conformation, whereas silica shell formation was used to prevent enzyme leakage by desorption, with a potential to provide increased pH and temperature resistance for the developed composites. 

**Scheme 1:**
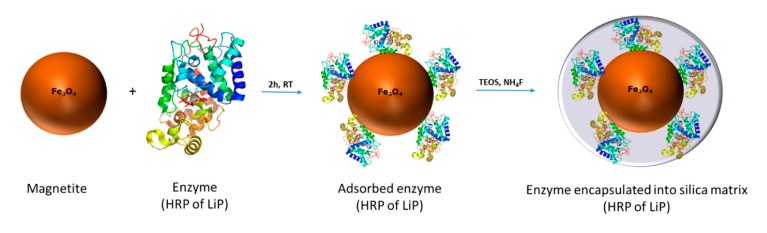
Sol–gel encapsulation of HRP- and LiP peroxidases adsorbed onto magnetite nanoparticles.

Synthesis was performed according to Scheme 1 Below: 

### 3.1. Transmission Electron Microscopy

**Figure 2:**
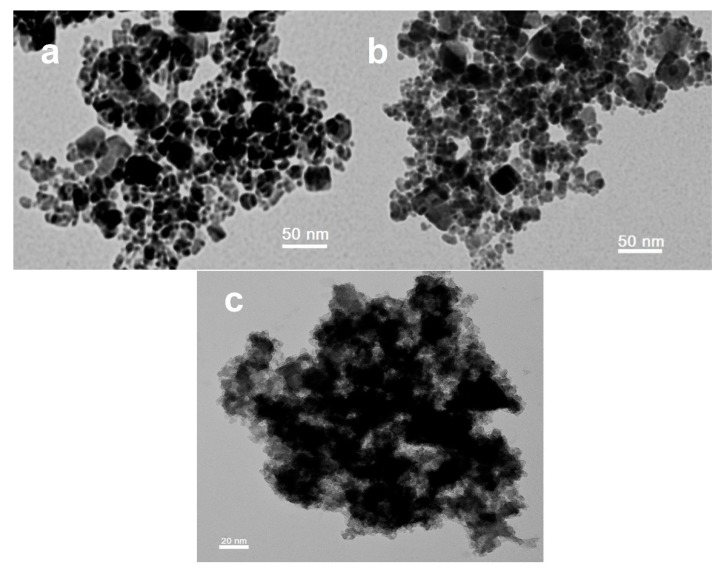
TEM images of magnetite (**a**), magnetite-LiP (**b**) and sol–gel encapsulated magnetite-LiP-silica composites (**c**).

Initial magnetite samples, magnetite with adsorbed LiP and silica-coated magnetite-LiP samples were characterized by transmission electron microscopy (Figure 2a–c, respectively). From the TEM images it was very clear seen that a silica shell was formed over the magnetite nanoparticles surface (Figure 2c). The original magnetite particles possessed the size 14 ± 3 nm, but partly were aggregated into blocks 30–40 nm in size. Their size and appearance did not change at all after enzyme grafting, because the enzyme molecules have much lower density and are essentially invisible in TEM. Coating of the material by silica results in rough aggregates of silica gel, incorporating the enzyme-grafted magnetite particles (darker areas in the Figure 2c). The silica shell has lower density (lighter in the TEM image). It can be seen that no non-coated magnetite particles remain in the material, proving successful encapsulation of the immobilized enzyme.

### 3.2. Atomic Force Microscopy

**Figure 3:**
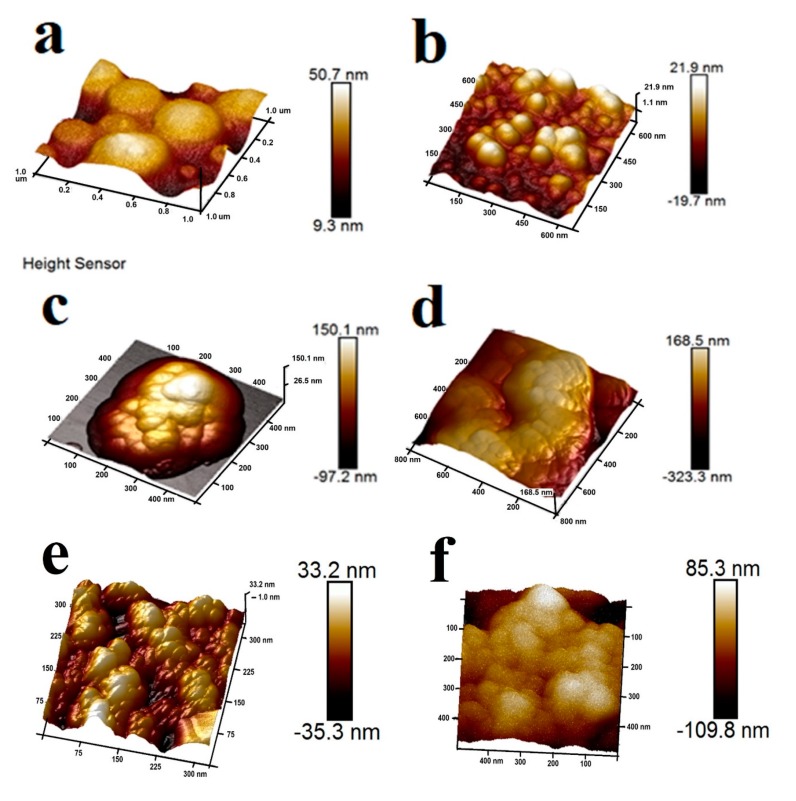
AFM images of native HRP (**a**) and LiP (**b**) enzymes and sol–gel encapsulated HRP (**c**) and LiP (**d**) composites and initial magnetite (**e**) and silica coated magnetite (**f**).

The textural characteristics of the enzyme-composites were investigated by AFM. Aggregated enzyme particles were visible on AFM images for native HRP- and LiP enzymes deposited on the surface (Figure 3a,b respectively). When immobilized, the enzymes were not evident on the surface of the magnetite, confirming that they were encapsulated and only aggregates of silica were visible on the surface (Figure 3c, respectively). Initial magnetite and silica-coated magnetite nanoparticles are shown in Figure 3e,f respectively. The AFM data was also in agreement with TEM analysis on synthesized composites (Figure 2). Since the appearance of pristine iron oxide and iron oxide bearing adsorbed enzymes does not differ significantly in TEM, a quantitative peak force measurement was carried out in AFM using the Bruker QPM software. It revealed very considerable difference in interaction of the cantilever with the two types of sample. The average adhesion force for pristine FeOx was 1.6 ± 0.5 nN, while for LiP coated particles the interaction was very much stronger with an average adhesion force of 80.8 ± 27.6 nN (see Figures S11 and S12). Coating with silica results in a rather uniform surface, causing strong variation in the background for the interaction force that decreases to a rather low adhesion value of 2.6 ± 1.2 nN.

### 3.3. Scanning Electron Microscopy

SEM images of the sol–gel HRP- and LiP samples are presented in Figure S1a–d respectively. The samples consisted of small particles below 1 µm and associated aggregates. 

### 3.4. Thermogravimetric Analysis

The total weight loss in sol–gel HRP- and LiP samples was 13.24% and 13.86% respectively (Figure S2a,b). In both samples, the mass loss below 100 °C was due to moisture evaporation while above 110 °C chemisorbed water evaporation as well as condensation of OH groups in silica layer occurs. Thermal destruction of the sol–gel encapsulated enzymes occurred in the temperature range 110–400 °C. The more exact temperature range for decomposition of the enzymes 220–450 °C was determined by DSC measurements (see Figure S14). 

### 3.5. FTIR

Figure S3 shows FTIR spectra for the synthesized sol–gel composites. Fe-O bond vibration from the magnetite core appears at 457 cm^−1^. Symmetrical and anti-symmetric vibrations of ν(Si-O-Si) appears at 800 cm^−1^ and 1000–1230 cm^−1^ and can be attributed to Si-O-Si vibrations in silica. Absorption band at 1630 cm^−1^ belongs to water molecule vibrations together with the broad absorption band 3000 cm^−1^ to 3600 cm^−1^.

### 3.6. Activity of the Composites

From the literature, it was reported that above pH 7.5 and below 4.0, LiP enzymes lose all activity in less than 5 h [38]. The native LiP half-life in solution at 25 °C was 198 h, whereas at 40 °C and pH 3.0, it losts its full activity. At pH 5 the activity was17%, while no activity was observed at pH 6 [39]. Free HRP undergoes activity loss at elevated temperatures, but when encapsulated is known to retain its activity even after 14 h at 55 °C [40]. The catalytic constant and efficacy increase after HRP immobilization on graphene oxide [41]. The authors explain the increased stability against pH change for immobilized HRP via action of the local buffering effect. For HRP, a broad range of optimal pH from 4 to 9 depending on the chemical nature of immobilization support have been reported [42]. 

**Figure 4:**
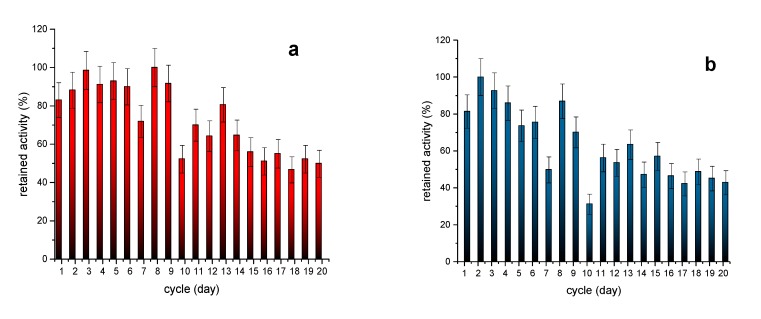
Activity of the composites in ABTS oxidation at 55 °C: HRP (**a**) and LiP (**b**).

Activity was evaluated using a model ABTS oxidation test. Each cycle was conducted for 24 h at 55 °C. As observed in Figure 4a,b, the composites retained their activity even after 20 consequent cycles at elevated temperature. The retained activity after 20th cycle was 43% for LiP and 50% for HRP. The gradual decrease in activity over time can be explained by enzyme deterioration by hydrogen peroxide. Poisoning of the enzyme active sites cannot be discounted. Nevertheless, encapsulated enzymes kept their activity much longer, than native LiP- and HRP enzymes that usually do not survive for even 24 h at elevated temperatures. 

### 3.7. Enzyme Leaching from Sol–Gel Encapsulated Composites

Enzyme leakage tests, were performed using both UV–Vis at λ = 280 nm to detect enzyme, and to evaluate possible enzymatic activity of leachate (ABTS, λ = 420 nm). Apparently, enzyme is gradually releasing from the composite (Figures S17 and S18). That is confirmed by protein absorption at λ = 280 nm, and also by enzymatic activity of leachate solutions. Enzyme leaching can explain decrease in composite activity, occurring with time.

### 3.8. Activity in Drug Decomposition and NMR Study of Decomposition Products

After testing ability of the composites in oxidation of model compounds, they were used for oxidation of several common water drug pollutants. It worth to notice that in our studies, at pH 7, at room and elevated temperatures (55 °C), the activity against selected drugs was negligible for both LiP and HRP composites. 

**Scheme 2:**
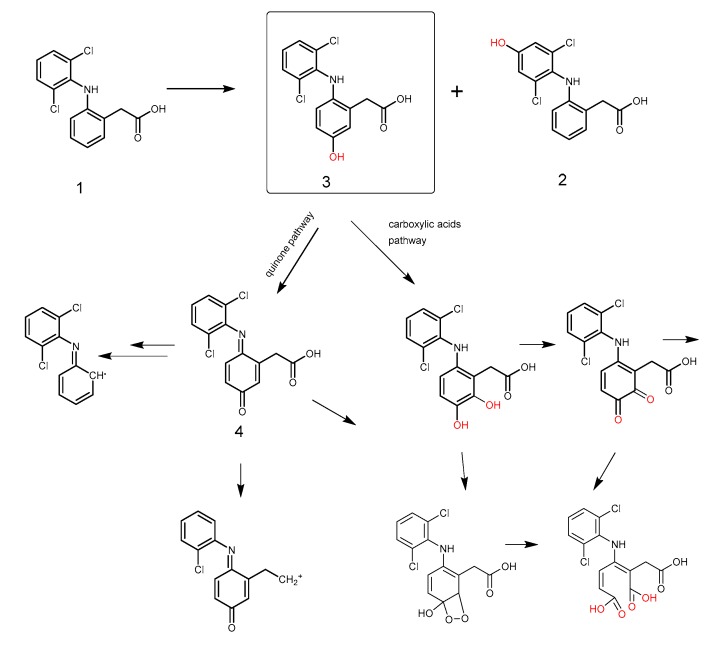
Possible pathways in diclofenac enzymatic oxidative decomposition.

Diclofenac is well known for its recalcitrance and difficulty for biodegradation [43,44,45] in nature. Destruction of diclofenac by oxidase enzymes apparently starts from hydroxylation of its molecule. For example, after laccase degradation two main metabolites were reported namely 4‘-hydroxy-diclofenac (compound **2**, Scheme 2) and 5-hydroxy-diclofenac (compound **3**, Scheme 2) [46]. Quinones may also be formed as diclofenac metabolites such as diclofenac-2,5-iminoquionone (compound **4**, Scheme 2). Nevertheless, it has been shown, that only 5–10% of the removed diclofenac was transformed into diclofenac-2,5-iminoquionone, suggesting that 90–95% was possibly oxidized via other pathways [47]. 

#### 3.8.1. Drug Degradation by Non-Encapsulated Enzymes 

Just adsorbed enzymes were evaluated in diclofenac and paracetamol degradation tests. Non encapsulated composites were active only in one cycle, after that enzyme was deactivated and washed out from the surface. Data presented on Figures S15 and S16.

#### 3.8.2. Degradation of Diclofenac by Non-Immobilized Enzymes 

The NMR spectra of initial diclofenac and diclofenac after interaction with native HRP and LiP enzymes are shown in Figure S4a–c respectively. Total degradation of diclofenac in the case of HRP is due to a higher number of enzyme units (10-fold number of enzyme units compared to LiP). Spectra b and c in Figure S4 are scaled 32 times. The chemical shifts for sodium diclofenac in ^1^H NMR were: 7.24 (d), 7.09 (t), 6.93 (t), 6.43 (d), 3.77 (s). 

#### 3.8.3. Degradation of Diclofenac by Sol–Gel Encapsulated Enzymes at pH 5

**Figure 5:**
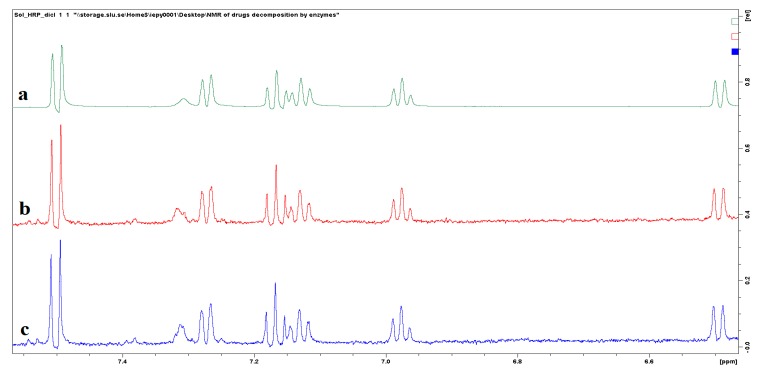
NMR spectra of initial diclofenac (**a**), diclofenac after interaction with sol–gel encapsulated LiP (**b**), and HRP (**c**) composites at pH = 5 during 3 days.

NMR spectra for diclofenac and its metabolites after interaction with sol–gel encapsulated HRP and LiP enzyme composites at pH 5 are shown in Figure 5a–c respectively. The spectra of diclofenac after interaction with both sol–gel encapsulated HRP and LiP composites are similar to each other. In comparison with initial diclofenac, new artefact peaks appear at 7.3–7.4 and at 7.55 ppm after interaction with composites. These peaks can be attributed to hydroxy-substituted derivatives of diclofenac. Spectra b and c in Figure 5 is scaled 32 times. 

#### 3.8.4. Degradation of Diclofenac by Sol–Gel Encapsulated Enzymes at pH 3

**Figure 6:**
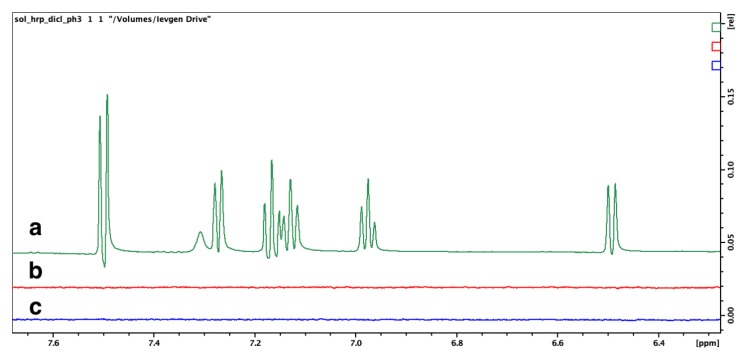
NMR spectra of initial diclofenac (**a**), diclofenac after interaction with sol–gel encapsulated LiP (**b**), HRP (**c**) composites at pH = 3 over 3 days.

From NMR spectra we can conclude that diclofenac removal by sol–gel encapsulated HRP and LiP enzymes at pH 3 lead to total degradation (see NMR on Figure 6a–c, respectively). In this example, we suppose that in addition to the quinone transformation pathway, the hydroxylated derivatives of diclofenac can undergo further oxidation to carboxylic acids with following breaking down of the aromatic ring (Scheme S1). Probably, products of degradation were insoluble and/or were adsorbed on composite surface.

The summarized pathways during enzymatic degradation of diclofenac is given on scheme below. 

#### 3.8.5. Degradation of Paracetamol by Non-Immobilized Enzymes

**Figure 7:**
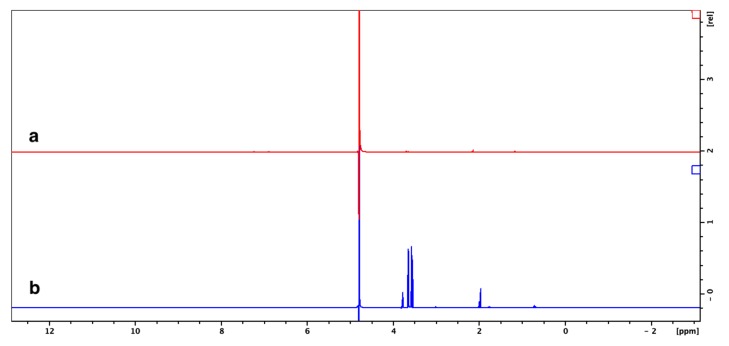
NMR spectra of paracetamol after interaction with HRP (**a**) and LiP (**b**) enzymes at pH = 7 during 5 days.

Chemical shifts for the paracetamol molecule in ^1^H NMR are the following: 9.5 (s), 6.9 (d), 7.2 (d), 2.1 (s). As can be observed form NMR spectra of paracetamol after interaction with native enzymes, aromatic protons, inherent initial paracetamol molecules, disappear (Figure 7). This leads us to conclude total degradation of the drug under the influence of enzymes.

#### 3.8.6. Degradation of Paracetamol by Sol–Gel Encapsulated Enzymes at pH 5

NMR spectra of initial paracetamol and its metabolites after interaction with sol–gel encapsulated HRP- and LiP composites at pH 5 are presented in Figure S5a–c, respectively. Simple conclusion can be done from these spectra, that paracetamol is not destroyed under pH 5 with both HRP and LiP composites.

#### 3.8.7. Degradation of Paracetamol by Sol–Gel Encapsulated Enzymes at pH 3

**Figure 8:**
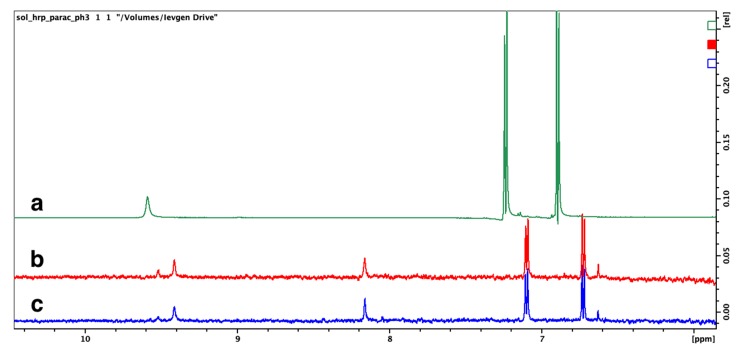
NMR spectra initial paracetamol (**a**), paracetamol after interaction with sol–gel HRP (**b**), and LiP (**c**) composites at pH = 3.

In contradiction to paracetamol degradation at pH 5, NMR profile of paracetamol metabolites at pH 3 is rather different. Except of decrease of concentration of paracetamol, signal from aromatic protons shifts. In addition, new signals at 8.18 ppm and 9.42 ppm started to appear on both HRP and LiP treated samples (Figure 8). Also, doublet of aromatic protons at 6.8–7.2, inherent for paracetamol, shifted right on 0.1 ppm, to 6.7–7.1 ppm in enzyme-treated samples. 

#### 3.8.8. Degradation of Carbamazepine by Non-Immobilized Enzymes

**Scheme 3:**
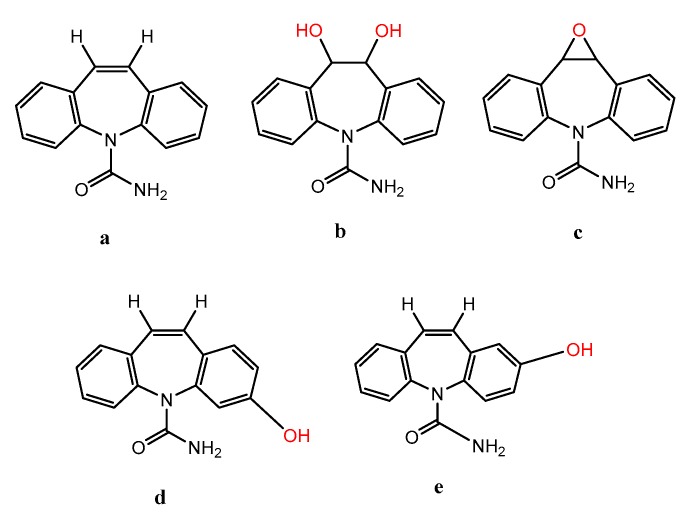
Chemical formula for CBZ (**a**) and its metabolites after enzymatic degradation (**b**–**e**).

The products after CBZ interaction with native HRP- and LiP enzymes are presented in Figure S6a–c respectively. A detailed mass-spectrometric investigation on the products for CBZ (Scheme 3, compound **a**) transformation by laccase is given in [48]. The two main products detected were: 10,11-dihydro-10,11-dihydroxy-CBZ (DiOH-CBZ) (Scheme 3, compound **b**) and 10,11-dihydro-10,11-epoxy-CBZ (EP-CBZ) (compound **c**). At the same time, major metabolites of CBZ formed by myeloperoxidase [49] were 2- and 3-OH metabolites of CBZ (Scheme 3, compounds **d** and **e**). Mechanisms for aromatic compound hydroxylation by enzymes are thoroughly discussed in [50].

Apparently, in case of native HRP and LiP enzymes interaction with CBZ, aromatic ring cleavage occurs, which reflects in disappearance of aromatic protons signal around 7.5 ppm.

#### 3.8.9. Degradation of Carbamazepine by Sol–Gel Encapsulated Enzymes at pH 5

In case of carbamazepine, only a decrease in concentration, but no metabolites were observed during decomposition by sol–gel encapsulated enzymes at pH 5 (see Figure S6a,d,e). Chemical shifts for CBZ in ^1^H NMR were: 7.4–7.27 (m), 6.90 (s), 4.95 (s).

#### 3.8.10. Degradation of Carbamazepine by Sol–Gel Encapsulated Enzymes at pH 3

At pH 3, total destruction of CBZ was observed. Possible products for CBZ during enzymatic degradation are not certain. 

**Figure 9:**
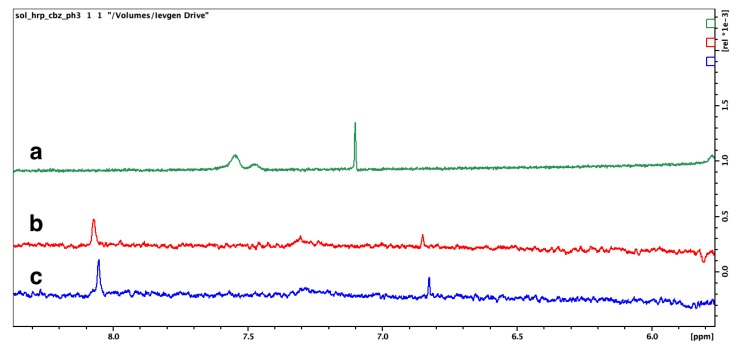
NMR spectra of initial CBZ (**a**), CBZ after interaction with sol–gel encapsulated LiP (**b**), and HRP (**c**) at pH = 3 over 3 days.

Since the aromatic protons in NMR spectra of CBZ products disappear (Figure 9a–c), it is likely that destruction of the aromatic CBZ moieties occurs. The mechanisms for CBZ transformations may also be different. If one considers that during peroxidase action, hydroxylated derivatives of CBZ are formed, then they may undergo further oxidation to carboxylic acids and quinones, involving aromatic ring cleavage, as was shown in Scheme 2. The signals at 6.8 and 8.05 ppm can be attributed to hydroxylated derivatives of CBZ.

The reason for increased enzymatic activity may be explained by literature data. For example, it was suggested [51] that the reason for activity enhancement of enzymes encapsulated within DNA scaffolds “was that the pH near the surface of the negatively charged DNA nanostructures was lower than that in the bulk solution, creating a more optimal pH environment for the anchored enzymes”. Furthermore, it is well-known that acidic catalysis increases performance of oxidation reactions, which can also be the reason for increased enzymes activity.

Results of decomposition for selected drugs by LiP and HRP composites under different conditions are summarized in Table 1.

**Figure 10:**
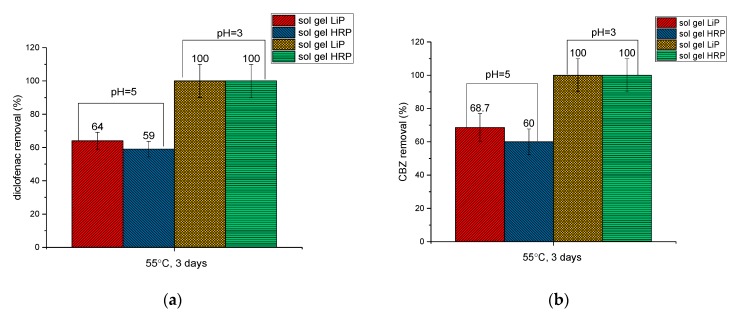
(**a**) Diclofenac removal at pH 5.0 and pH 3.0. (**b**) CBZ removal at pH 5.0 and pH 3.0.

To summarize, a decrease in pH appreciably increased the enzymes activities. Changing pH to 5.0 increased the activity of the composites allowing them to decompose more than 50% of diclofenac (Figure 10a) and CBZ within 3 days (Figure 10b). In the case of paracetamol, the decomposition rate was below 10% (NMR in Figure S7).

An explanation for poor paracetamol degradation may lie in a different mechanism of oxidation. In contrast to laccases, peroxidases are heme-containing enzymes, which utilize hydrogen peroxide when oxidized releasing two electrons. Then the peroxidase is reduced via a two-step one-electron oxidation process. Lignin peroxidase (LiP, E.C. 1.11.1.14) oxidizes phenolic compounds and reduces molecular oxygen to water, generating intermediate radicals. These radicals are different from those generated by laccase, resulting in different pollutant degradation mechanisms for the two enzymes. Transformation of polyphenols, such as lignin, to low-molecular compounds is the most frequent reaction of LiP in nature [52]. For example, LiP oxidizes and degrades lignin (natural polyphenol) according to Scheme 2 [52,53,54]. The main mechanism is formation of carboxylic acids and subsequent ring cleavage and macromolecule breakdown. The scheme for phenol transformation by peroxidase in presence of hydrogen peroxide is presented on Scheme S1. When compared to laccase, where phenoxy-radicals are formed, peroxidases generate cation-radicals (Scheme S1). Phenoxy-radicals produced by laccase result in high levels of paracetamol removal due to polymerization. In the case of peroxidases, cation-radicals apparently formed more effectively with CBZ and diclofenac than on paracetamol, with subsequent molecule degradation and removal. However, intermolecular radical scavenging in the case of paracetamol should not be discounted.

Lower oxidation efficiencies for immobilized enzymes can be attributed to an increased mass transfer requirement and limited accessibility of active sites after enzyme immobilization [55,56]. At pH 3 and 55 °C, total decomposition of diclofenac and carbamazepine was observed (Figure 10a,b, and discussion of NMR studies), in the case of both HRP- and LiP composites. Nevertheless, paracetamol was not totally degraded by immobilized enzymes even at this low pH (see NMR in Figure S7). 

## 4. Conclusions

Peroxidase enzymes HRP and LiP, efficiently immobilized on iron oxide nanoparticles by adsorption from solution, maintain their active conformation. Adsorption is especially visible in quantitative measurements of adhesion by AFM. The advantage of the proposed approach was in potential preservation of advantageous configuration of enzyme molecules when adsorbed on magnetite nanoparticles, which was confirmed by further tests. Further sol–gel coating by silica results in permanent immobilization with resistance to ‘washing off’ and enhanced stability in time and against elevated temperatures and acidity. Synthesized composites were active in diclofenac, carbamazepine, and paracetamol removal. Immobilized LiP was more active in diclofenac removal than HRP and even laccase, as was anticipated based on earlier studies. The silica-coated enzyme composites revealed enhanced stability at elevated temperatures. Nevertheless, the silica encapsulated HRP- and LiP enzyme composites showed limited ability for paracetamol degradation. In addition, cation-radicals apparently formed by peroxidase enzymes, were more effective for CBZ and diclofenac removal than in case of paracetamol. 

## Figures and Tables

**Table 1:** Activity of LiP and HRP composites in drug decomposition processes over 3 days, 55 °C at different pHs.

*pH = 5*			*pH = 3*	
	Sol–Gel Fe_3_O_4_/HRP/SiO_2_,	Sol–Gel Fe_3_O_4_/LiP/SiO_2_	Sol–Gel Fe_3_O_4_/HRP/SiO_2_,	Sol–Gel Fe_3_O_4_/LiP/SiO_2_
Diclofenac	59% ± 8%	64% ± 8%	100% ± 10%	100% ± 10%
Paracetamol	9% ± 5%	9% ± 5%	50 ± 10%	50 ± 10%
Carbamazepine	60% ± 8%	68 ± 8%	100% ± 10%	100 ± 10%

## Supplementary Materials

The following are available online at https://www.mdpi.com/2079-4991/10/2/282/s1, Figure S1: SEM images of sol gel encapsulated HRP (a,b) and LiP (c,d) composite samples, Figure S2: TGA of freeze-dried HRP sample (a) and LiP sample (b), Figure S3: FTIR spectra of the sol gel HRP (a) and sol gel LiP (b) comsposite samples and enzyme-free sol-gel silica (c), Figure S4: NMR spectra of initial diclofenac (a), diclofenac after interaction with sol gel encapsulated LiP (b), and HRP (c) composites at pH = 5 during 3 days, Figure S5: NMR spectra of initial carbamazepine (a), CBZ after interaction with native LiP enzyme (b), HRP enzyme (c), at pH = 7 during 5 days, and CBZ after contact with sol gel encapsulated LiP (d) and HRP (e) at pH = 5, Figure S6: NMR spectra initial paracetamol (a), paracetamol after interaction with sol gel HRP (b) and LiP (c) composites at pH = 5, Figure S7: NMR spectra of initial diclofenac (d), diclofenac after interaction with sol gel encapsulated LiP (e), HRP (f) composites at pH = 3 during 3 days, Figure S8: NMR spectra of initial CBZ (a), CBZ after interaction with sol gel encapsulated LiP (b), and HRP (c) at pH = 3 during 3 days, Figure S9: NMR spectra initial paracetamol (a), paracetamol after interaction with sol gel HRP (b) and LiP (c) composites at pH = 3, Figure S10: NMR spectra of diclofenac (a), diclofenac after interaction with native LiP (b) and HRP (c) enzymes during at pH = 7 during 5 days, Figure S11: Representative image of a peak force curve for the pristine iron oxide particles, Figure S12: Representative image of a peak force curve for the LiP coated iron oxide particle, Figure S14: DSC data for Fe_3_O_4_/HRP/SiO_2_ (a) and Fe_3_O_4_/LiP/SiO_2_ composites (b), Figure S15: Paracetamol degradation non-encapsulated HRP, pH = 7 (t = 18 h, C = 1 mg/ml), Figure S16: Diclofenac degradation non-encapsulated HRP, pH = 7 (t = 19 h 40 min, C = 1 mg/ml), Figure S17: Normalized enzyme activity of leachate solution (a) and optical density of leachate solution (b) for Fe_3_O_4_/HRP/SiO_2_ composite, Figure S18: Normalized enzymatic activity of leachate solution (a) and optical density of leachate solution (b) for LiP composites, Scheme S1: Chemical formula for CBZ and its metabolites after enzymatic degradation.
