# Supplementary material for: Removal of Diclofenac, Paracetamol, and Carbamazepine from Model Aqueous Solutions by Magnetic Sol–Gel Encapsulated Horseradish Peroxidase and Lignin Peroxidase Composites
Source: Nanomaterials (Basel). 2020 Feb 7;10(2):282. doi: 10.3390/nano10020282 (PMC7075194; doi:10.3390/nano10020282)
Supplement: Supplementary file 1 [file nanomaterials-10-00282-s001.pdf]

nanomaterials

Supplementary Material

Removal of Diclofenac, Paracetamol, and Carbamazepine from Model Aqueous Solutions by Magnetic Sol–Gel Encapsulated Horseradish Peroxidase and Lignin Peroxidase Composites

Ievgen V. Pylypchuk [1,2,*], Geoffrey Daniel [3], Vadim G. Kessler [1], and Gulaim A. Seisenbaeva [1,*]

[1] Department of Molecular Sciences, Swedish University of Agricultural Sciences, 750 07 Uppsala, Sweden; vadim.kessler@slu.se
[2] Chuiko Institute of Surface Chemistry of National Academy of Sciences of Ukraine, 17, General Naumov Street, 03164 Kyiv, Ukraine
[3] Department of Biomaterials and Technology, Swedish University of Agricultural Sciences, Box 7008, 750 07 Uppsala, Sweden; geoffrey.daniel@slu.se
* Correspondence: ievgenpy@kth.se (I.V.P.); gulaim.seisenbaeva@slu.se (G.A.S.)

Received: 28 December 2019; Accepted: 01 February 2020; Published: 7 February 2020

Supplementary Figures

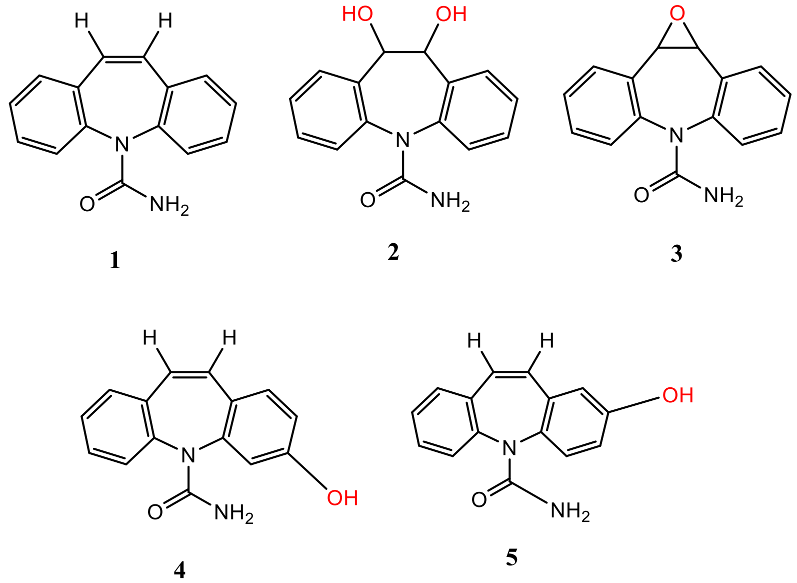

Scheme S1. Chemical formula for CBZ (1) and its metabolites after enzymatic degradation (2-5).

Scanning electron microscopy (SEM).

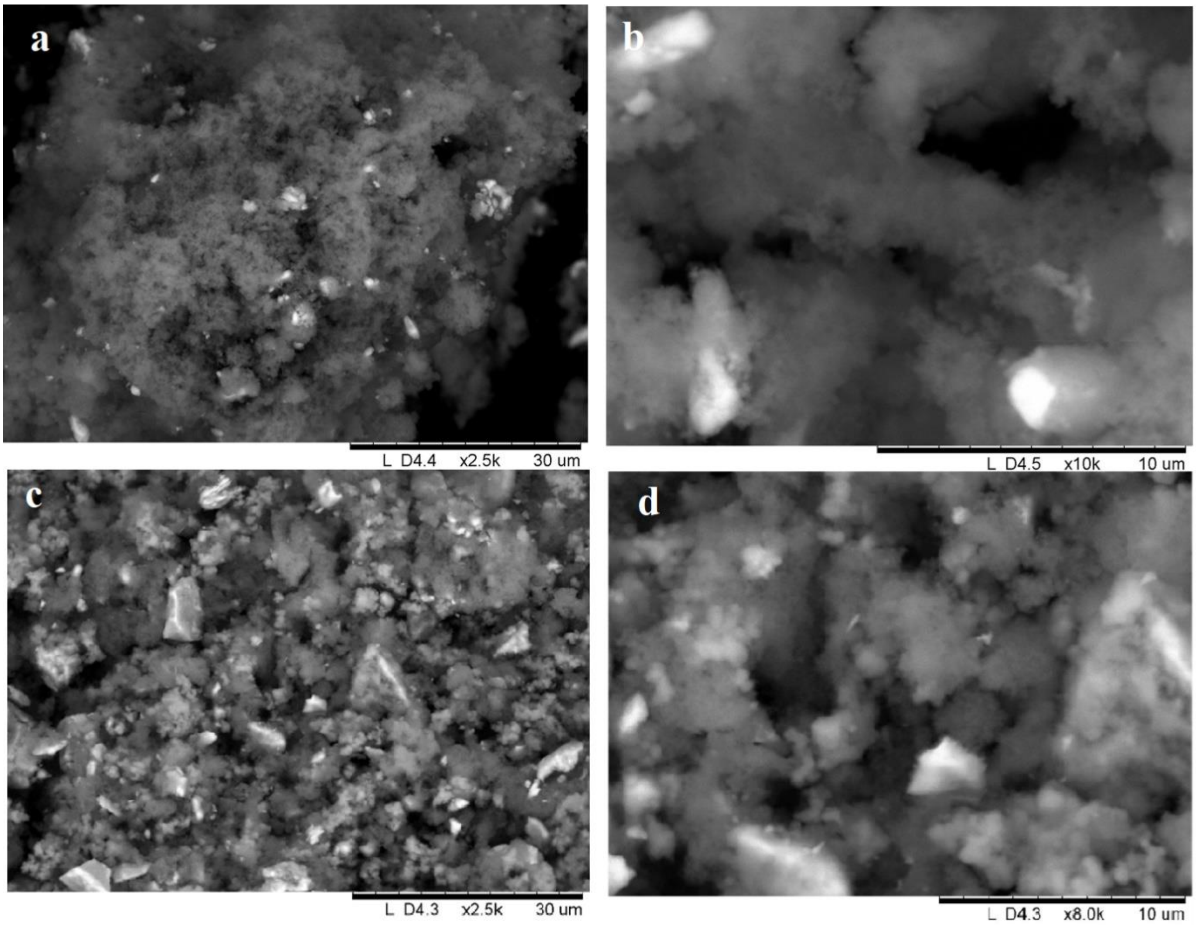

Figure S1. SEM images of sol gel encapsulated HRP (a,b) and LiP (c,d) composite samples.

Thermogravimetric analysis.

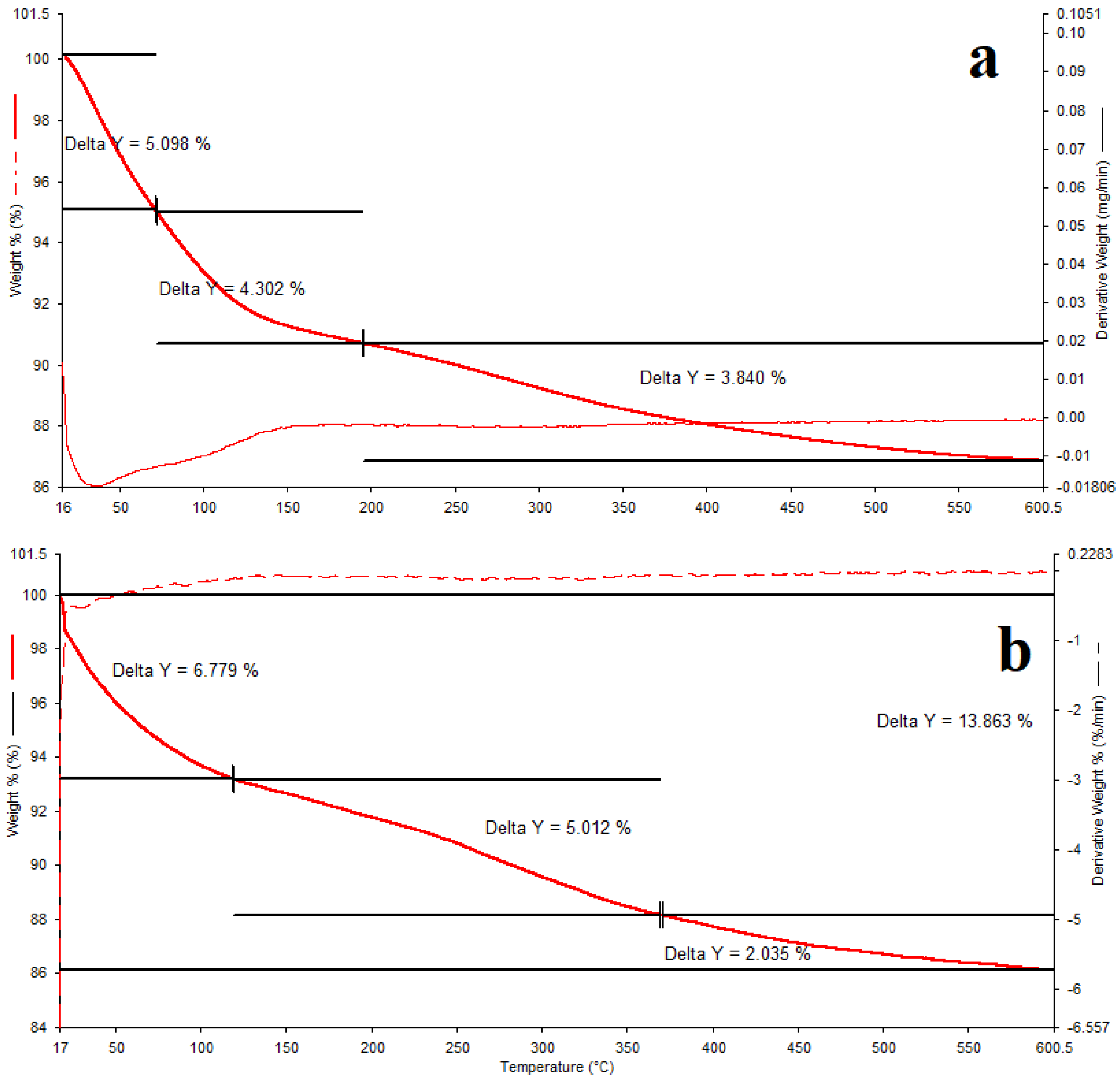

Figure S2. TGA of freeze-dried HRP sample (a) and LiP sample (b).

FTIR.

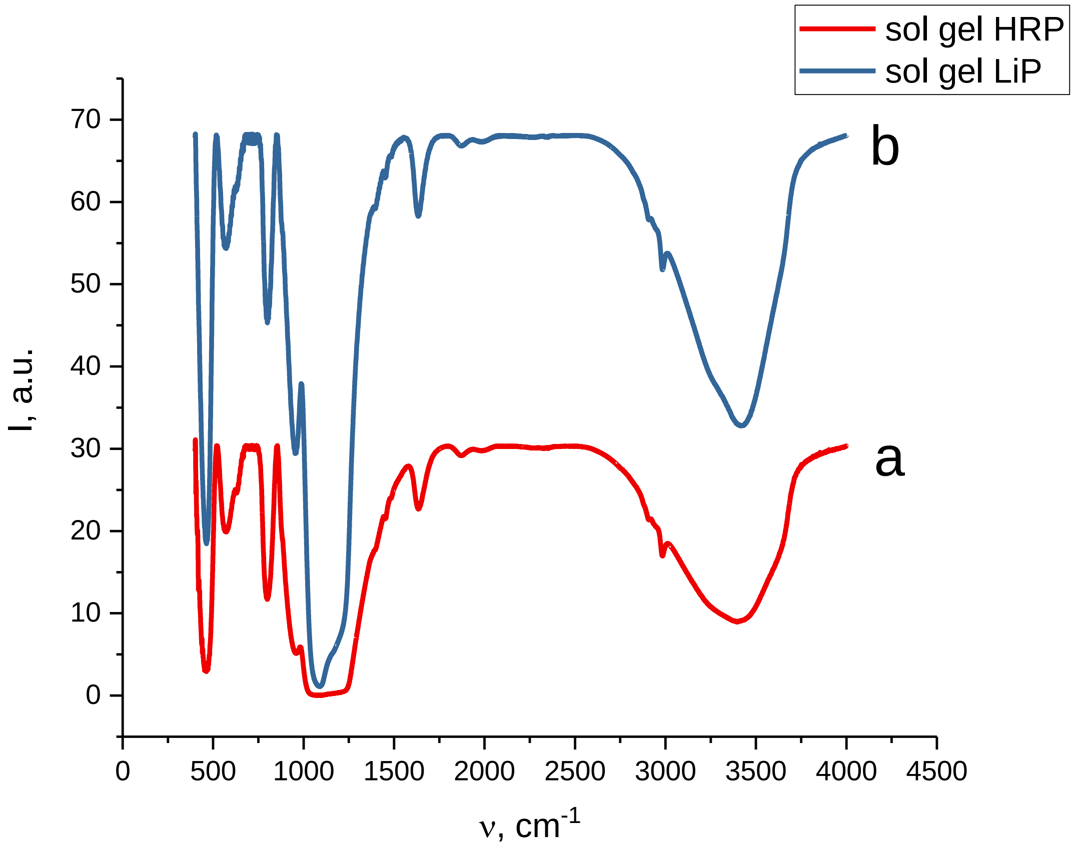

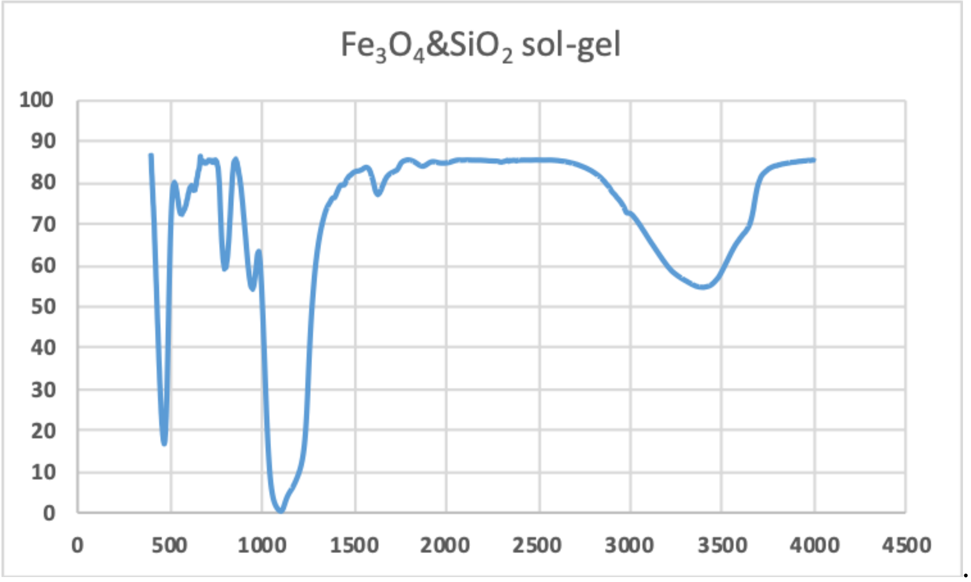

Figure S3. FTIR spectra of the sol gel HRP (a) and sol gel LiP (b) comsposite samples and enzyme-free sol-gel silica (c). It has to be noted that the spectra are identical, showing only characteristic bands of sol-gel silica:.

Assignments for FTIR below.

472 vs δ (Si-O-Si)
806 s ν (Si-O)
952 s ν (Si-OH)

1117-1230 vvs ν (Si-O-Si)
1637 w δ (O-H)in Si-OH
1735 sh δ (OH) adsorbed water
3000 w, sharp ν (O-H) from Fe-OH
3417 s br ν (O-H) from Si-OH
3636 sh ν (O-H) adsorbed water

NMR.

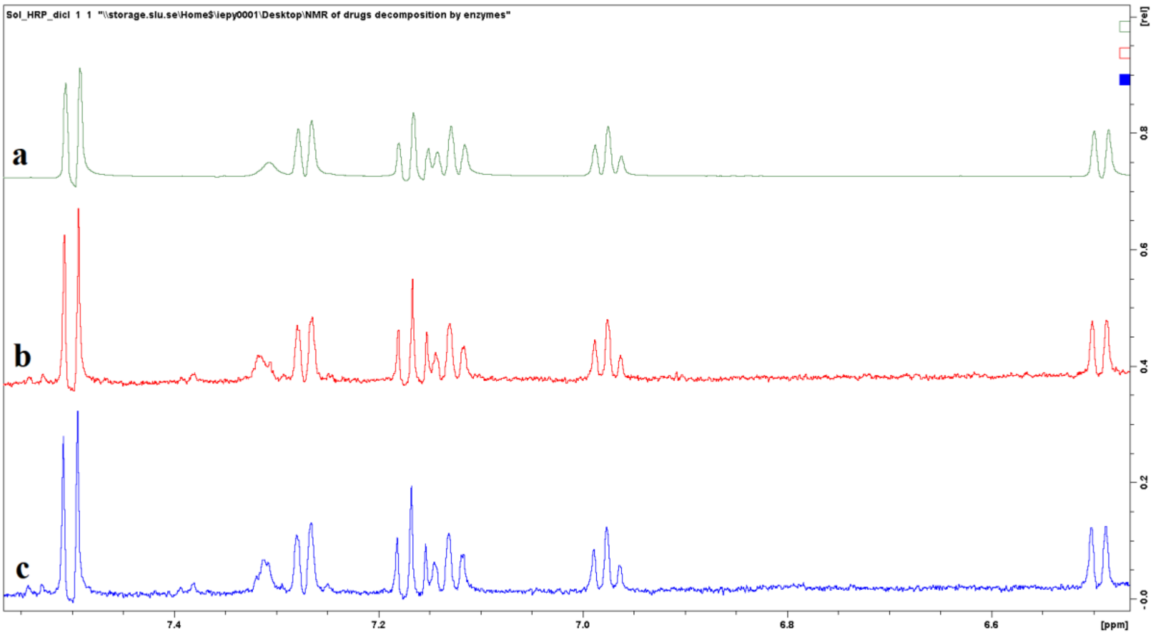

Figure S4. NMR spectra of initial diclofenac (a), diclofenac after interaction with sol gel encapsulated LiP (b), and HRP (c) composites at pH=5 during 3 days.

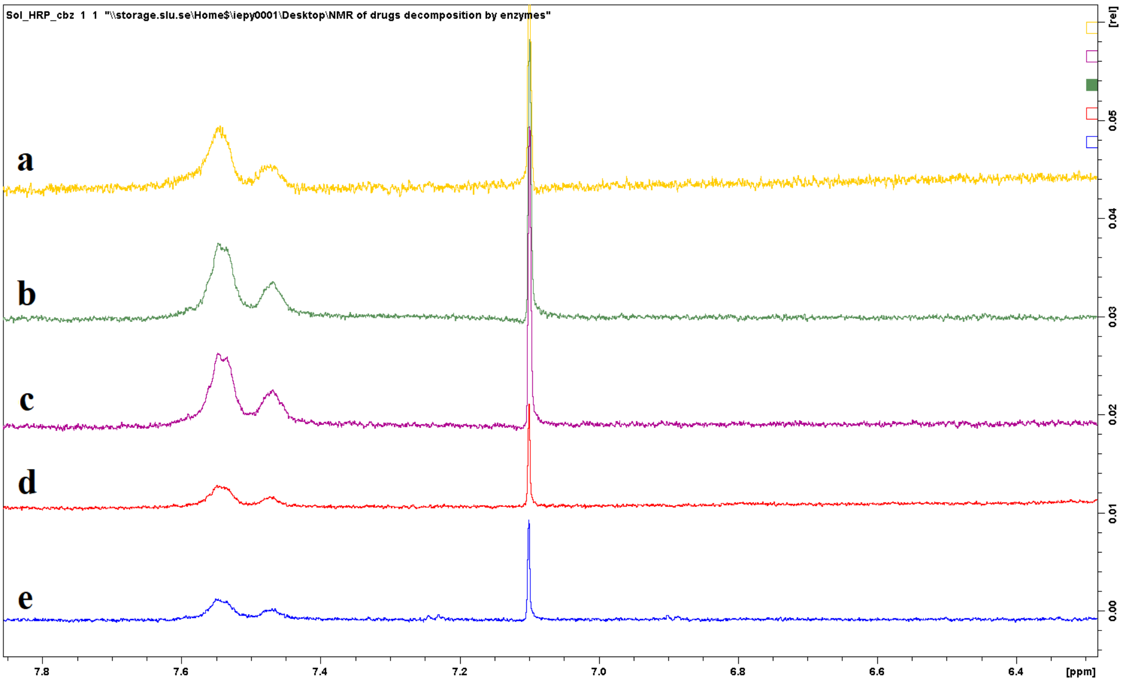

Figure S5. NMR spectra of initial carbamazepine (a), CBZ after interaction with native LiP enzyme (b), HRP enzyme (c), at pH=7 during 5 days, and CBZ after contact with sol gel encapsulated LiP (d) and HRP (e) at pH=5.

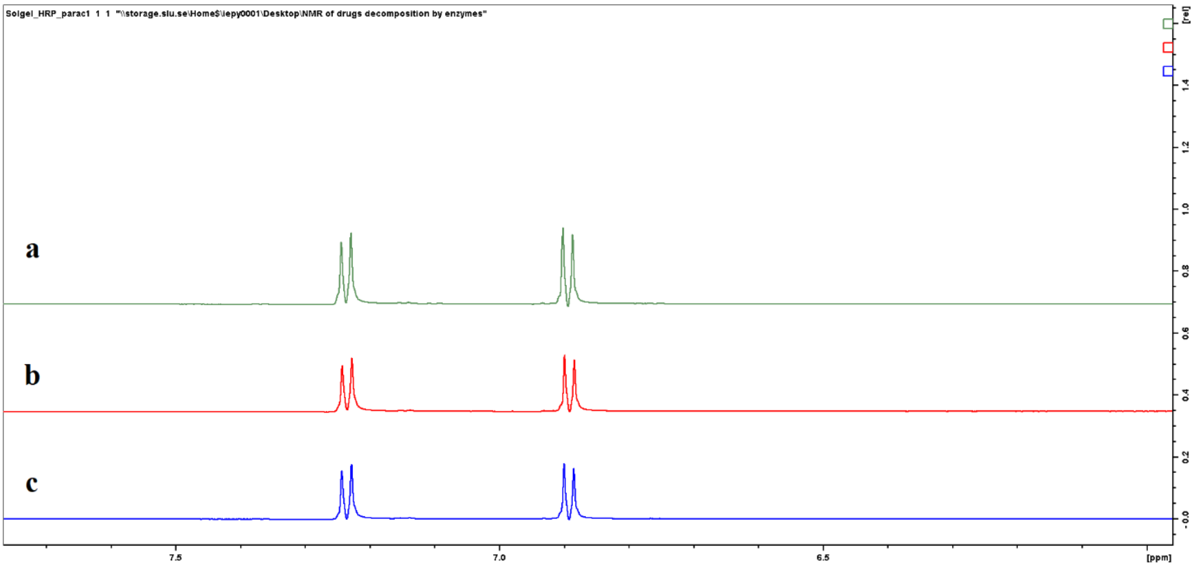

Figure S6. NMR spectra initial paracetamol (a), paracetamol after interaction with sol gel HRP (b) and LiP (c) composites at pH=5.

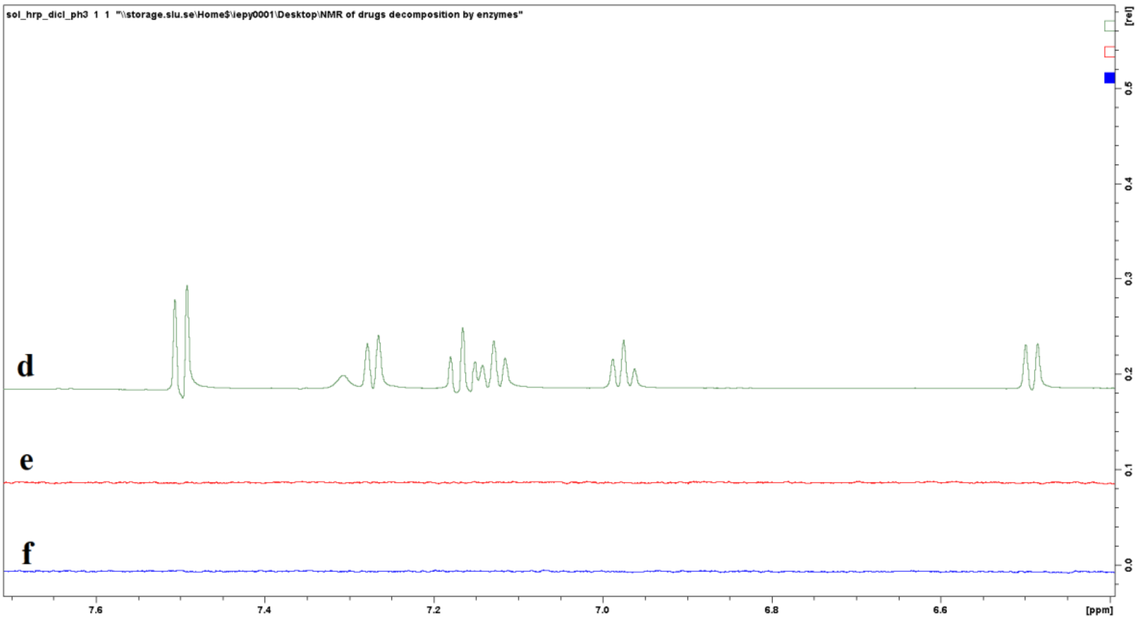

Figure S7. NMR spectra of initial diclofenac (d), diclofenac after interaction with sol gel encapsulated LiP (e), HRP (f) composites at pH=3 during 3 days.

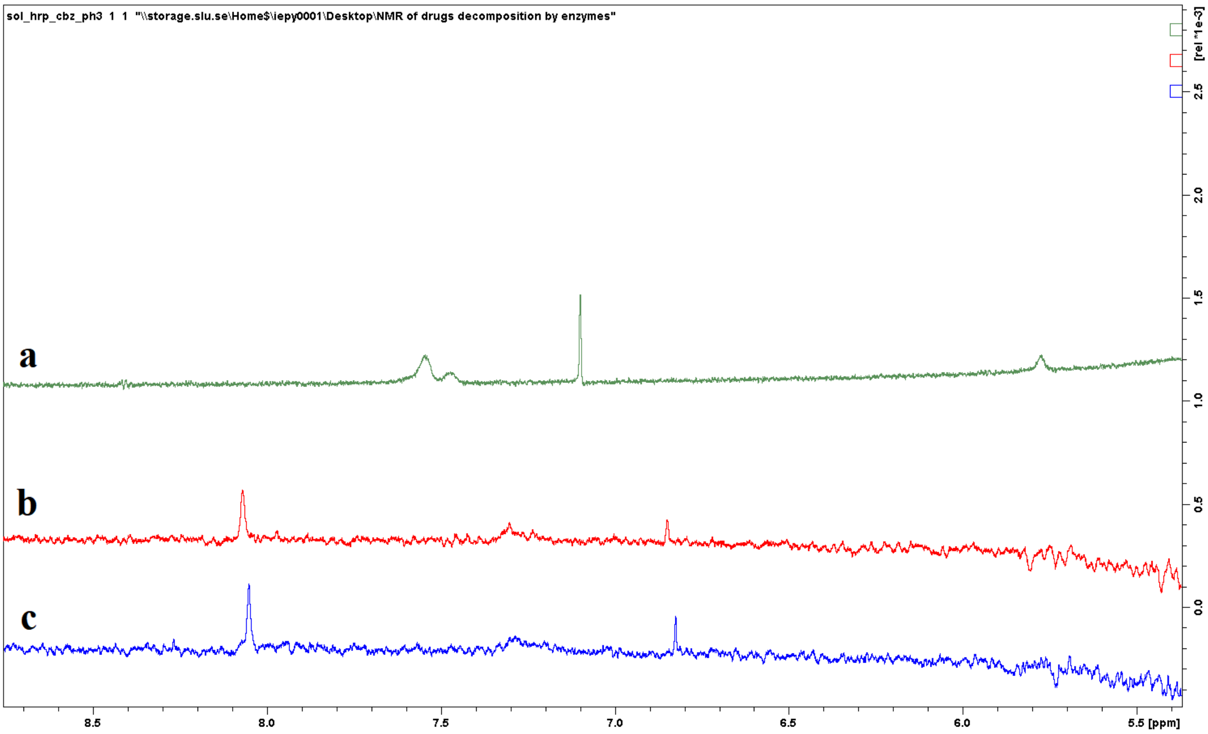

Figure S8. NMR spectra of initial CBZ (a), CBZ after interaction with sol gel encapsulated LiP (b), and HRP (c) at pH=3 during 3 days.

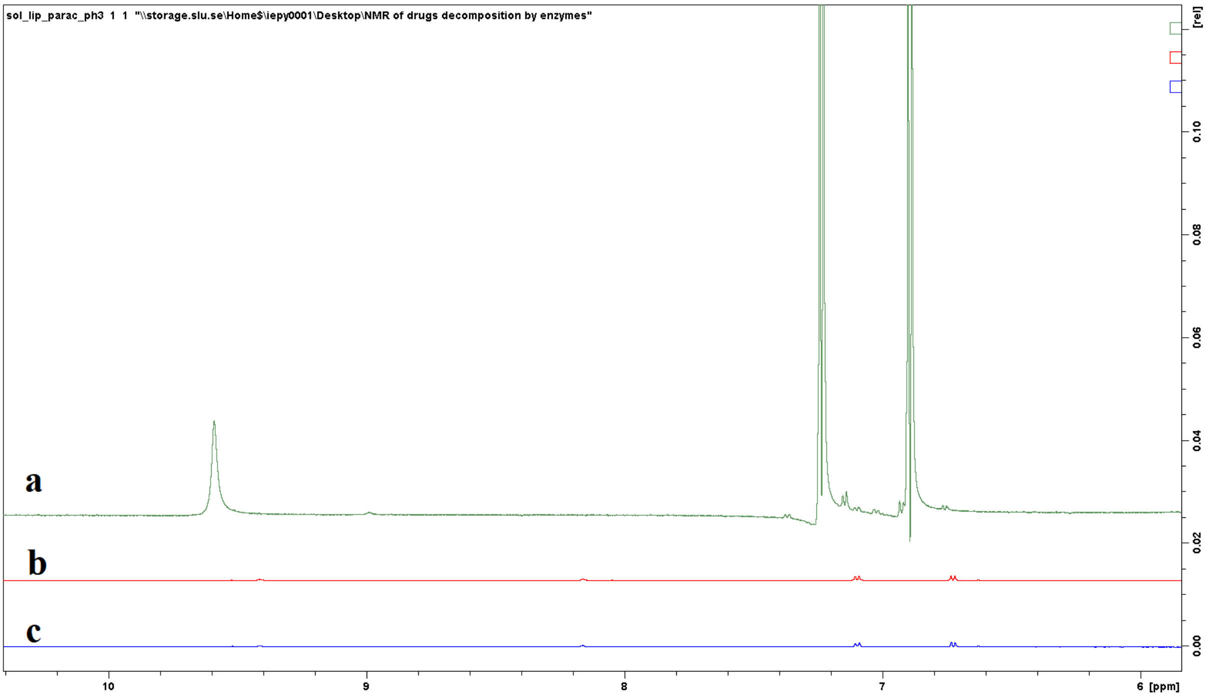

Figure S9. NMR spectra initial paracetamol (a), paracetamol after interaction with sol gel HRP (b) and LiP (c) composites at pH=3.

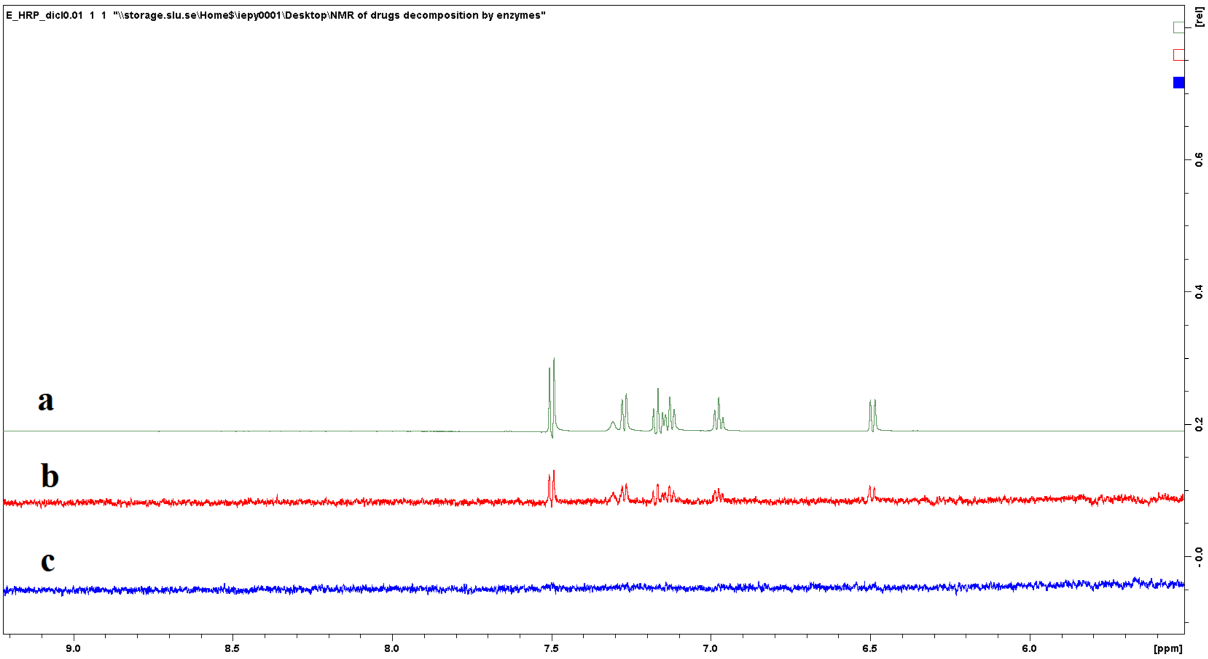

Figure S10. NMR spectra of diclofenac (a), diclofenac after interaction with native LiP (b) and HRP (c) enzymes during at pH=7 during 5 days.

AFM data.

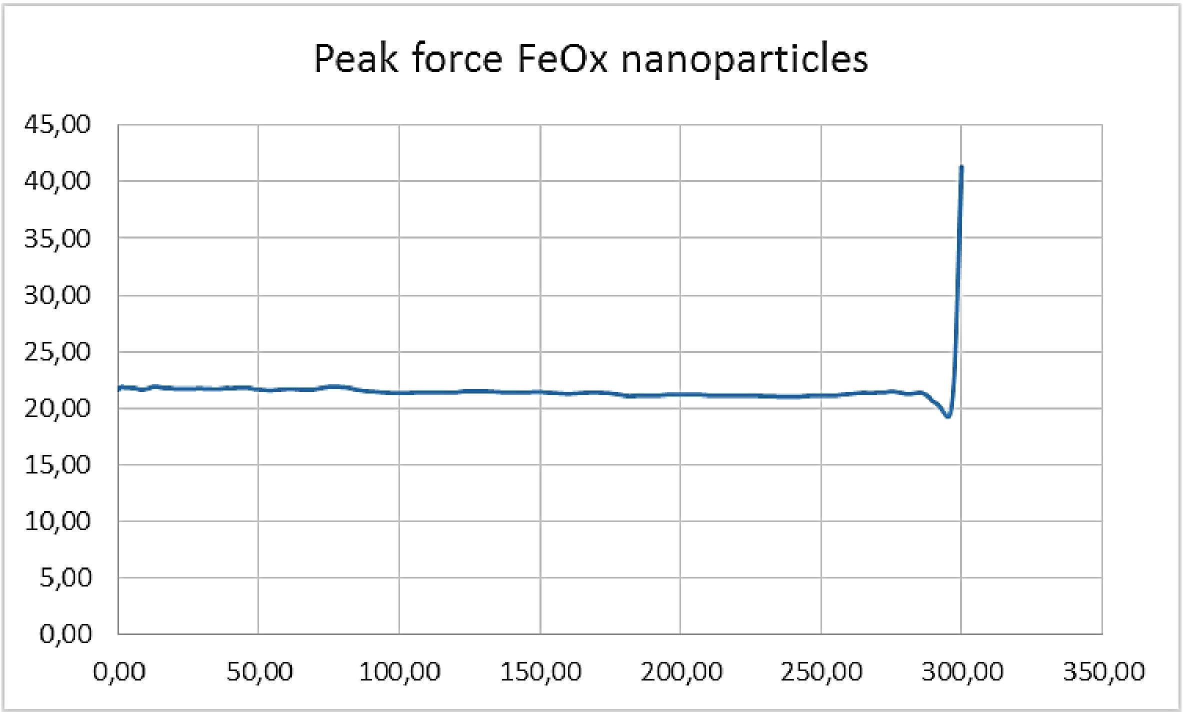

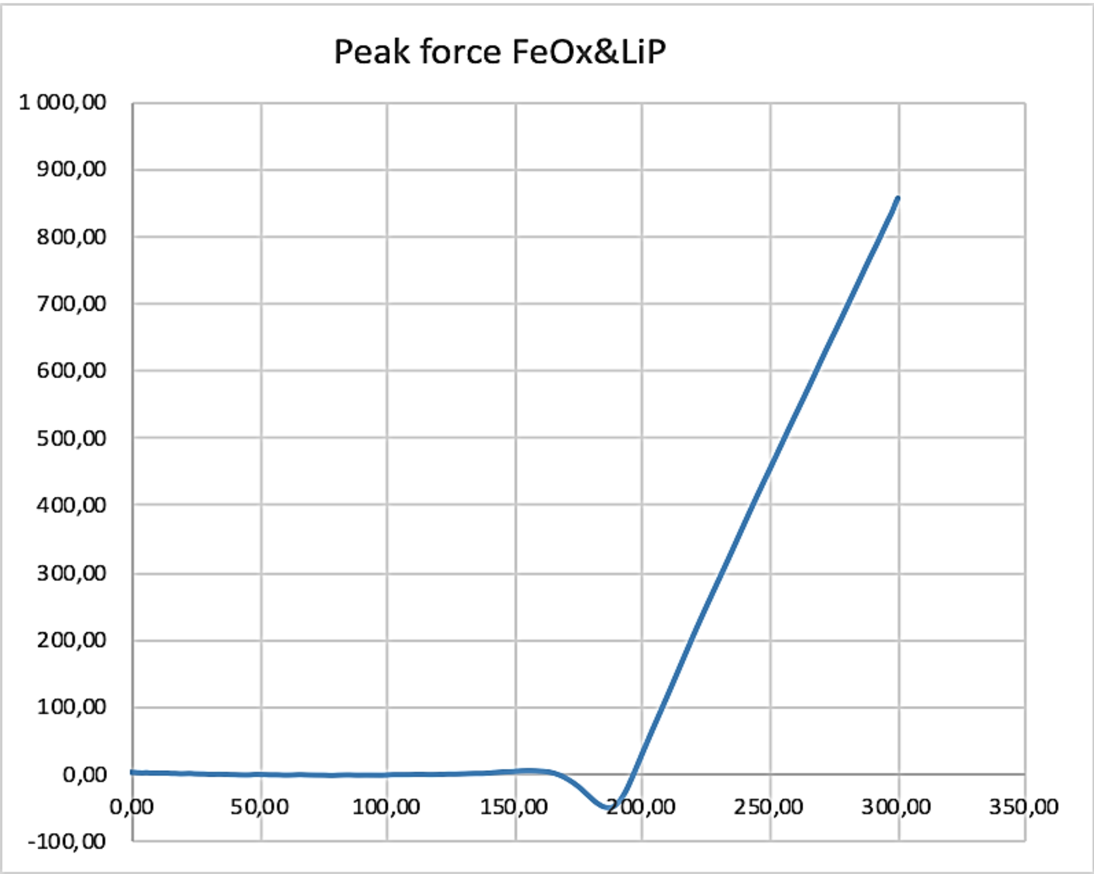

Figure S12. Representative image of a peak force curve for the LiP coated iron oxide particle.

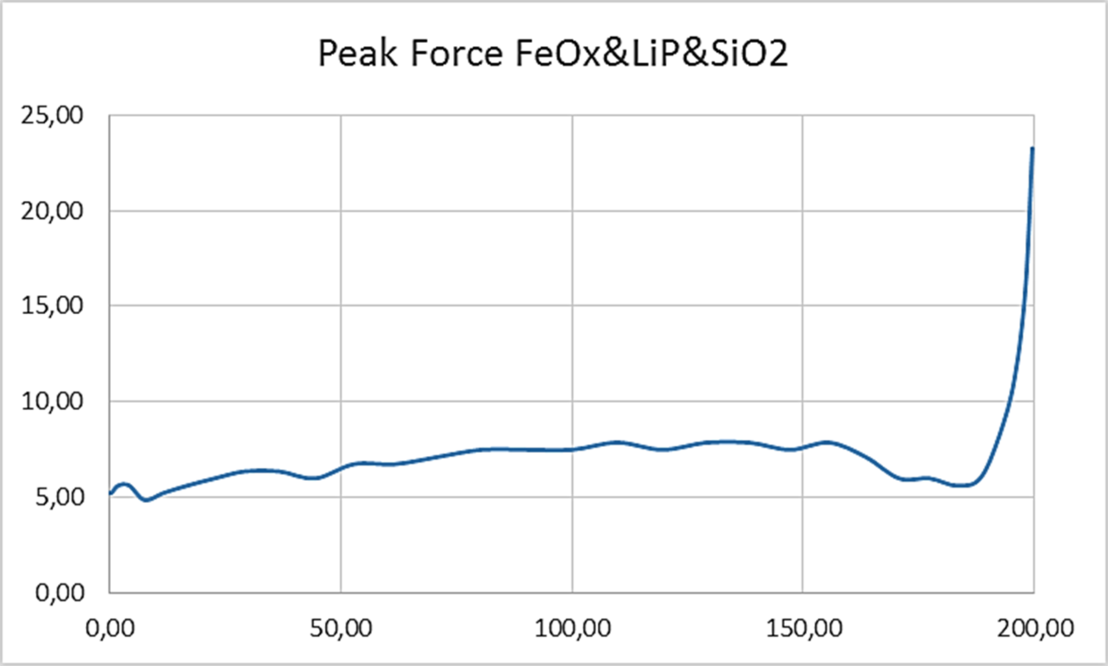

Figure S13. Representative image of a peak force curve for the silica-coated LiP-bearing iron oxide particles.

Differential scanning calorimetry.

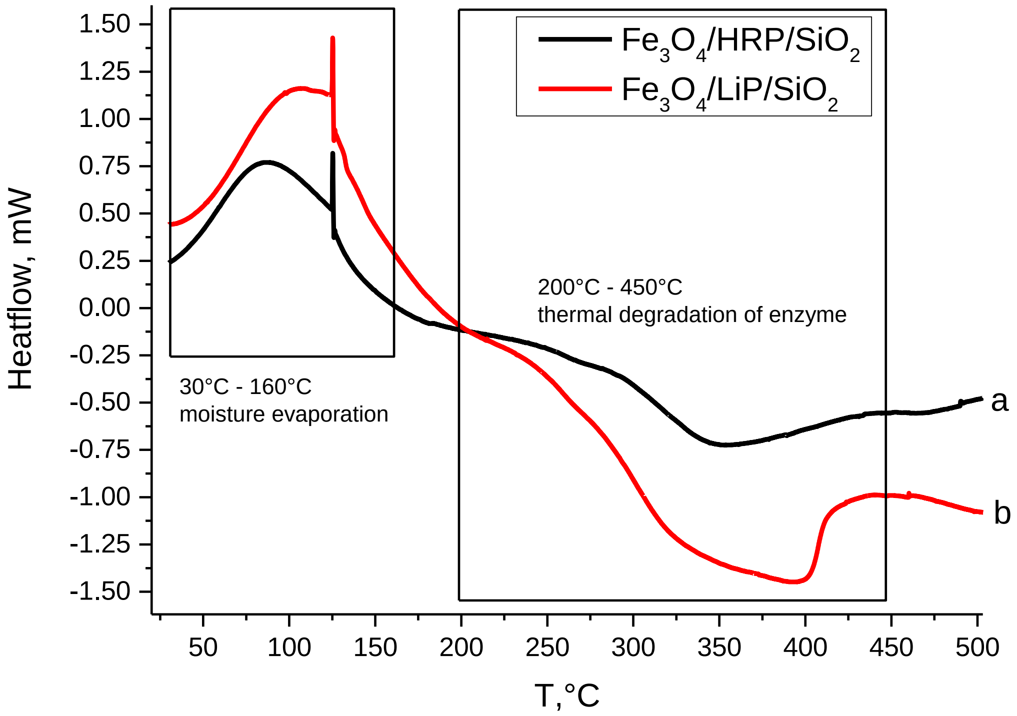

Figure S14. DSC data for Fe_3O_4/HRP/SiO_2 (a) and Fe_3O_4/LiP/SiO_2 composites (b).

Drugs degradation by non-encapsulated enzymes.

Experiments were done with- and without enzymes; experiments were multiplicated and ran in a few cycles.

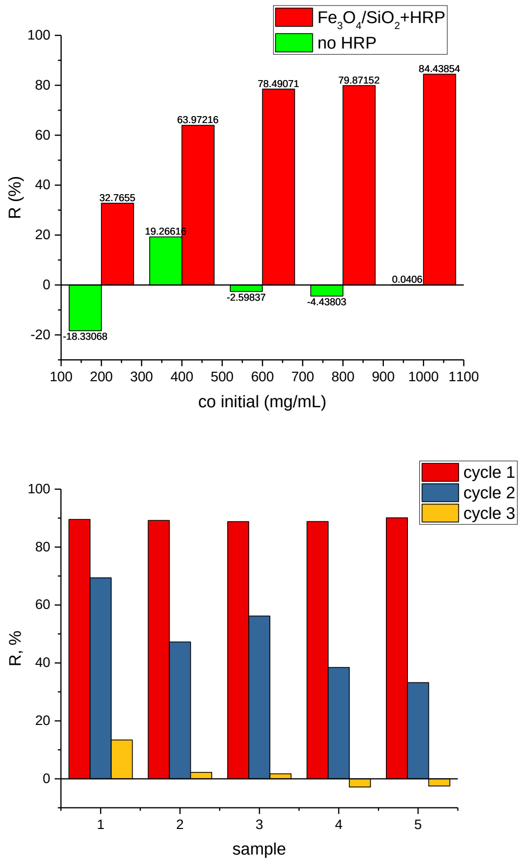

Figure S15. Paracetamol degradation non-encapsulated HRP, pH=7 (t=18 h, C=1 mg/ml).

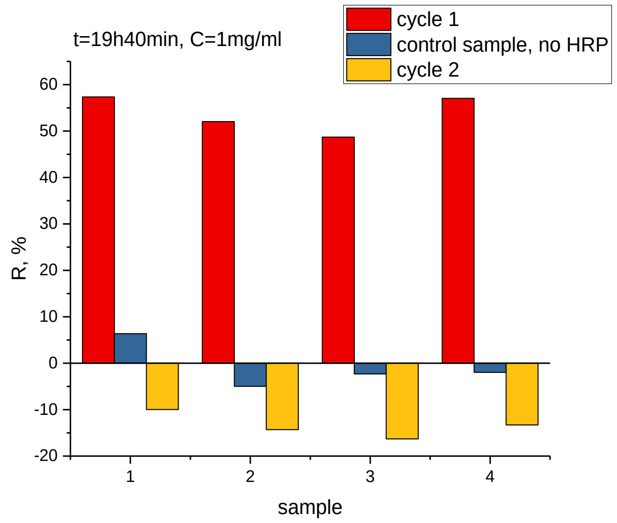

Figure S16. Diclofenac degradation non-encapsulated HRP, pH=7 (t=19h40 min, C=1mg/ml).

Enzyme leaching from sol gel encapsulated composites.

Enzyme leakage tests, were performed using both UV-Vis at λ = 280 nm to detect enzyme, and to evaluate possible enzymatic activity of leachate (ABTS, λ = 420 nm). Tests were performed as following. 30 mg of sample has been shaking at 55 °C for 24 h in deionized water. After composite decantation by magnet, solutions were filtered through 200 μm membrane filter and measured by UV-Vis at λ = 280 nm. Then, ABTS was added and solution was kept for 24 h at 55 °C to react. Activity was measured at λ = 420 nm. In total, 12 measurements were done. Data is shown below.

Normalized optical density shown in negative values for better representation.

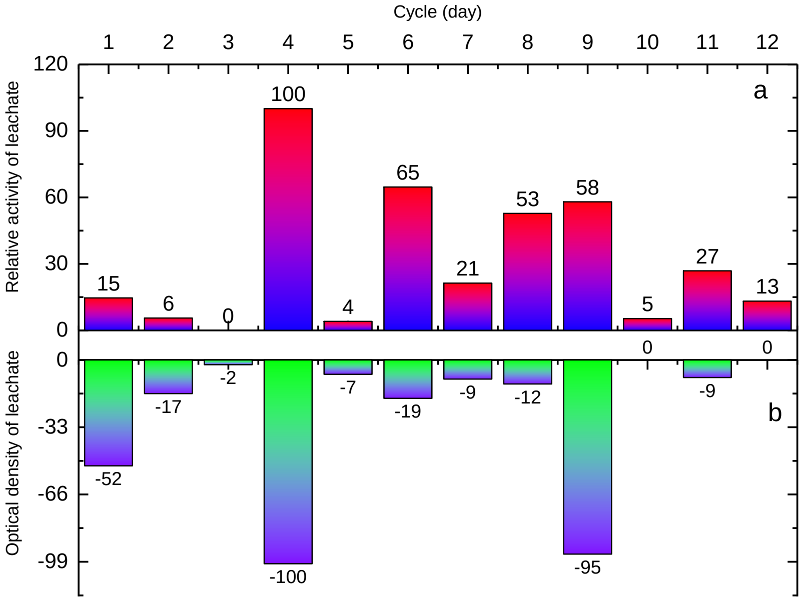

Figure S17. Normalized enzyme activity of leachate solution (a) and optical density of leachate solution (b) for Fe_3O_4/HRP/SiO_2 composite.

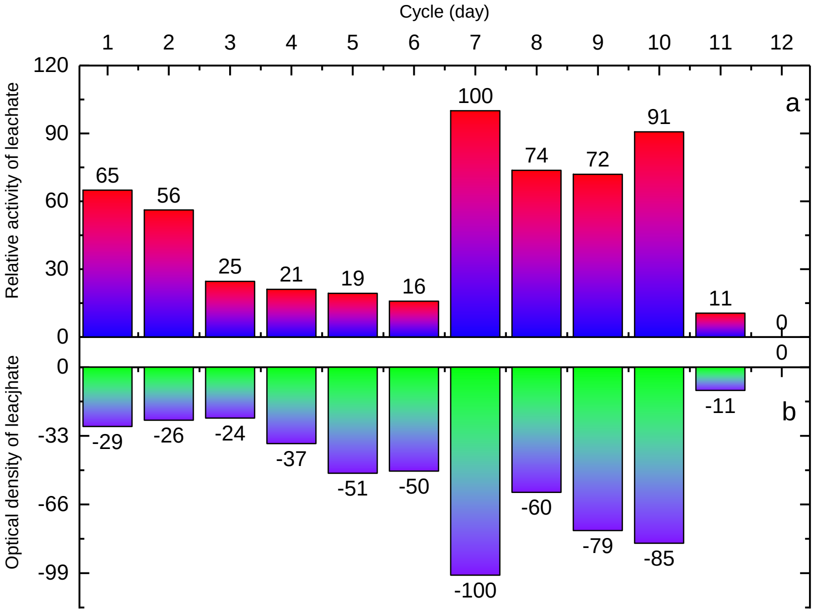

Figure S18. Normalized enzymatic activity of leachate solution (a) and optical density of leachate solution (b) for LiP composites.
